# Mesenchymal Stem Cell Transplantation Increases Antioxidant Protein Expression and Ameliorates GP91/ROS/Inflammasome Signals in Diabetic Cardiomyopathy

**DOI:** 10.3390/jcdd9110381

**Published:** 2022-11-07

**Authors:** Wei-Syun Hu, Tung-Sheng Chen, Ka-Hung Cheang, Wei-Yu Liao, Chin-Hsien Chang

**Affiliations:** 1 School of Medicine, College of Medicine, China Medical University, Taichung 40042, Taiwan; 2 Division of Cardiovascular Medicine, Department of Medicine, China Medical University Hospital, Taichung 40447, Taiwan; 3 Graduate Program of Biotechnology and Pharmaceutical Industries, National Taiwan Normal University, Taipei 11677, Taiwan; 4 Traditional Chinese Medicine Department, En Chu Kong Hospital, New Taipei City 40237, Taiwan; 5 Department of Cosmetic Science, Chang Gung University of Science and Technology, Taoyuan 33303, Taiwan; 6 College of Chinese Medicine, China Medical University, Taichung City 40402, Taiwan

**Keywords:** cardiomyopathy, diabetes, inflammasome, mesenchymal stem cells, reactive oxygen species

## Abstract

Background: Cardiomyopathy is one of the complications associated with diabetes. Due to its high prevalence, diabetic cardiomyopathy has become an urgent issue for diabetic patients. Various pathological signals are related to diabetic cardiomyopathy progress, including inflammasome. Mesenchymal stem cell transplantation is full of potential for the treatment of diabetic cardiomyopathy because of stem cell cardiac regenerative capability. This study investigates whether mesenchymal stem cell transplantation shows therapeutic effects on diabetic cardiomyopathy through inflammasome signaling regulation. Methods: Wistar male rats were divided into three groups including Sham, T1DM (rats with type 1 diabetes) and T1DM + WJSC (T1DM rats receiving 1 × 10^6^ stem cells per rat). Results: Compared to the Sham, experimental results indicated that several pathological conditions can be observed in heart tissues with T1DM, including structural change, fibrosis, oxidative stress elevation and inflammasome related protein expression. All of these pathological conditions were significantly improved in T1DM rats receiving mesenchymal stem cell transplantation (T1DM + WJSC). Furthermore, the experimental findings suggest that mesenchymal stem cell transplantation exerted antioxidant protein expression in diabetic heart tissues, resulting in a decrease in oxidative stress and inflammasome signaling blockage. Conclusion: These findings imply that mesenchymal stem cell transplantation shows therapeutic effects on diabetic cardiomyopathy through inflammasome regulation induced by oxidative stress.

## 1. Introduction

Clinically, diabetes is defined as patients with a fasting blood glucose over 200 mg/dL and HbA1c (glycated hemoglobin) over 6.5% [1]. Based on statistical reports, there will be approximately 6.9 billion people suffering from diabetes in the year 2045 [2]. One third of this diabetic population will be affected by cardiovascular diseases [3]. It is noteworthy that cardiac dysfunction will become an urgent issue to be solved in patients with diabetes. Diabetic cardiomyopathy is one of the heart dysfunctions induced by diabetes. Among diabetic patients, 16.9% meet the criteria for diabetic cardiomyopathy [4]. Several pathological signals are related to diabetic cardiomyopathy progress, including the production of reactive oxygen species [5], deposition of fibrotic collagen [6], hypertrophy of the left ventricle [7] and cardiomyocyte apoptosis [8]. In addition to the above signals, inflammasome pathway activation is also mentioned in diabetic cardiomyopathy.

The inflammasome pathway can be triggered by proinflammatory mediators, such as LPS [9]. Several proteins play central roles in inflammasome pathway activation, including the Nod-like receptor family pyrin domain-containing 3 (NLRP3), caspase-1, IL-1 beta and IL-18. The beginning of inflammasome activation produces the NLRP3/caspase-1 complex. The NLRP3/caspase-1 complex then matures into pro-interleukins (pro-IL-1 beta and pro-IL-18) as interleukins. The matured interleukins function as proinflammatory mediators to positively increase the inflammatory response, leading to cell damage [10]. Peng et al. [11] illustrated that inflammasome related proteins, including NLRP3, caspase-1, IL-1 beta and IL-8, are highly expressed in diabetic cardiomyopathy. Furthermore, inflammasome pathway activation is positively associated with pathological signaling activation, such as fibrosis, hypertrophy, apoptosis and pyroptosis, leading to diabetic cardiomyopathy progress [12,13].

A stem cell is a kind of cell that exhibits stemness, including self-renewal, paracrine and differentiation. A number of studies illustrated that stem cells show therapeutic effects on diabetic cardiomyopathy. Da Silva et al. [14] stated that stem cells ameliorate diabetic cardiomyopathy progress via anti-apoptotic, anti-fibrotic and anti-inflammatory effects. Graneli et al. [15] pointed out that stem cells show a protective effect on diabetic cardiomyopathy through proliferative capability and differentiation potential. Linthout et al. [16] confirmed that the immunomodulatory, anti-oxidative and pro-angiogenic features of stem cells play central roles in the treatment of diabetic cardiomyopathy. This study investigates whether mesenchymal stem cell transplantation shows a therapeutic effect on diabetic cardiomyopathy through inflammasome pathway regulation. The detailed mechanism between stem cell therapy and inflammasome regulation in the treatment of diabetic cardiomyopathy will be discussed in this study.

## 2. Materials and Methods

### 2.1. Chemicals and Reagents

All chemicals used in this study were purchased from Sigma-Aldrich Inc. (St. Louis, MO, USA). GP91, Sirt-1, SOD2, IL-1 beta, NLRP3, Caspase-1 and GAPDH antibodies for performing western blotting analysis were purchased from Thermo Fisher Scientific (Waltham, MA, USA).

### 2.2. Culturing Wharton’s Jelly Mesenchymal Stem Cells (WJSC)

WJSC were obtained from Professor CY Huang Lab (Cardiovascular and Mitochondrial Related Diseases Research Center, Hualien Tzu Chi Hospital, Buddhist Tzu Chi Medical Foundation, Hualien, Taiwan). The culture medium for WJSC was purchased from Thermo Fisher Scientific (Waltham, MA, USA) containing Dulbecco’s modified Eagle’s medium, 10% fetal bovine serum, 100 U/mL penicillin, 100 μg/mL streptomycin and 2 mM L-glutamine. Stem cells were incubated in CO_2_ incubator 37 °C under 5% CO_2_.

### 2.3. Animal Model

Twenty-four male Wistar rats (10 weeks-old, 200–250 g, BioLASCO Taipei, Taiwan ) were adapted in an animal room in a 12 h light–dark cycle with 25 °C of ambient temperature for 2 weeks. Rats were then divided into three groups including Sham (rats injected normal saline), T1DM (rats injected 50 mg/kg of streptozotocin via intraperitoneal route with fasting blood glucose level higher than 200 mg/dL) and T1DM+WJSC (T1DM rats receiving 1 × 10^6^ WJSC/rat through intravenous route). All experimental rats were sacrificed after receiving WJSC for 2 months. The heart tissues were isolated and preserved at −80 °C in a freezer for further study. The protocol for animal study was approved by the Institute Animal Care and Use Committee of National Taiwan Normal University (Protocol number 109030).

### 2.4. Hematoxylin and Eosin (HE) Stain

Deparaffinized and rehydrated heart tissue slides were used to performed HE stain in accordance with the manufacturer’s protocol (H&E staining kit, Waltham, MA, USA). After staining, the heart tissue slides were placed and observed with a microscope with a CCD camera. The images for stained tissue were saved for further investigation.

### 2.5. Masson’s Trichrome Stain

Deparaffinized and rehydrated heart tissue slides were used to perform Masson’s Trichrome stain based on a commercial kit (Trichrome stain kit, Waltham, MA, USA). After staining, fibrotic fiber showed as stained with blue color. The blue fiber deposition level was proportional to the tissue fibrosis level.

### 2.6. Thiobarbituric Acid Reactive Substance (TBARS) Assay

TBARS level measurements for blood samples were conducted using a commercial kit (TBARS assay kit, Waltham, MA, USA). Briefly, blood samples were mixed with TBA buffer and incubated in a water bath (95 °C) for 60 min. n-butanol was then added to TBA-sample solution and mixed well. After mixing, the n-butanol layer (pink color) was transferred onto 96-well plates. The optical density was read at the 532 nm wavelength.

### 2.7. Western Blotting Analysis

Homogenized heart tissues (40 μg) were transferred to 12% SDS separating gels with constant voltage (75 V). The separating gels were then placed with polyvinylidene difluoride (PVDF) membranes under constant voltage (50 V) for 1.5 h. PVDF membranes were then soaked in tris-buffered saline containing 3% bovine serum albumin. After blocking, primary and secondary antibodies were applied to PVDF membranes and the blotting bands visualized with Fujifilm LAS-3000 (GE Healthcare).

### 2.8. Statistics

All data were expressed as mean ± standard deviation (SD) and the significance for groups was analyzed with one-way analysis of variance. Statistical significance is considered when *p* < 0.05.

## 3. Results

### 3.1. Structural Analysis for Experimental Heart Tissues

**Figure 1:**
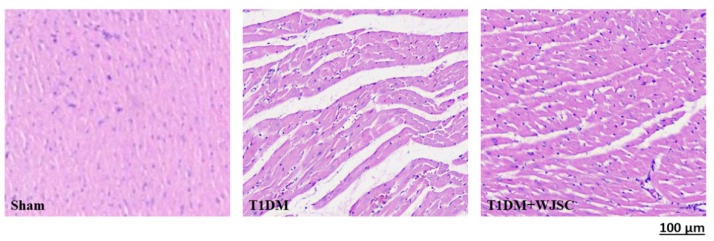
HE stain for heart tissues.

**Figure 2:**
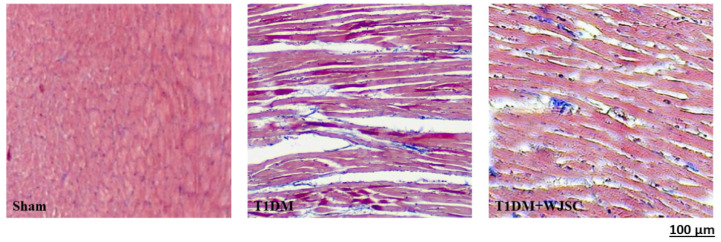
Masson’s Trichrome stain for heart tissues.

The heart tissue damage results in changes in the cardiac structure. Figure 1 shows the HE stain for experimental heart tissues. Compared to the Sham, the T1DM group exhibited extracellular space, cardiomyocytes disarray and immune cells infiltration. After stem cell therapy (T1DM + WJSC), the aforementioned pathological observations were significantly improved when compared to T1DM. Figure 2 illustrates the blue fiber deposition using Masson’s Trichrome stain. The blue fiber deposition level is proportional to the cardiac fibrosis level. We can see the blue fiber deposition in the T1DM group is more significant than that in the Sham. By contrast, stem cell therapy (T1DM + WJSC) significantly reduced the blue fiber deposition compared to T1DM.

### 3.2. Oxidative Stress Measurement for Experimental Heart Tissues

**Figure 3:**
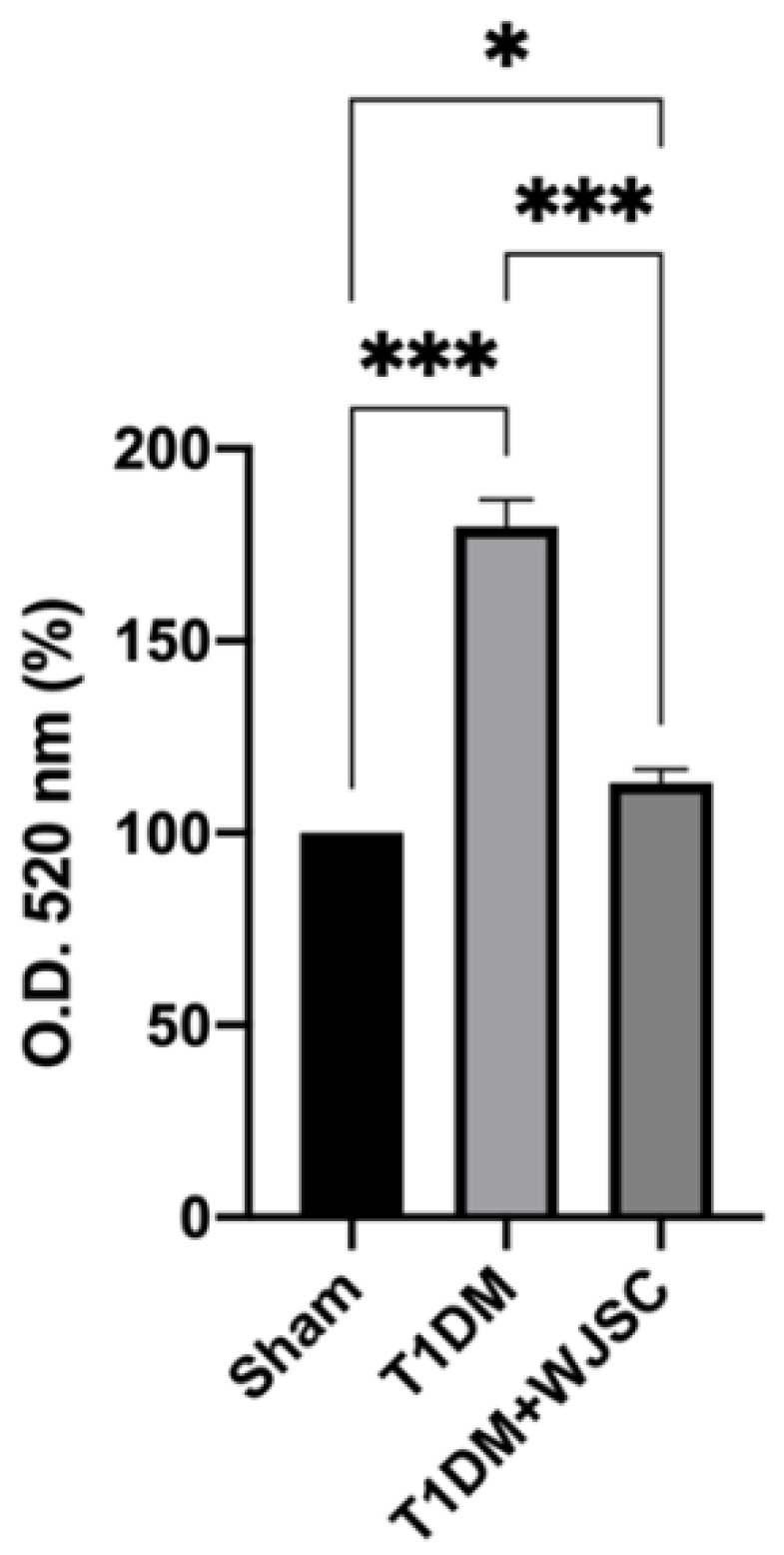
TBARS levels determination for heart tissues. * *p* < 0.05; *** *p* < 0.001.

TBARS are stable byproducts after ROS oxidize lipids. Thus, the quantity of TBARS is positively associated with the oxidative stress level. Figure 3 shows the TBARS level for experimental heart tissues. The TBARS levels for the Sham, T1DM and T1DM + WJSC follow the order 100 ± 0, 179 ± 7.2 and 113 ± 3.4, respectively. Significance can be observed between the Sham vs. T1DM (Sham < T1DM, *p* < 0.001) and T1DM vs. T1DM + WJSC (T1DM > T1DM + WJSC, *p* < 0.001).

### 3.3. Investigating Oxidative Stress Related Protein Expression Using Western Blotting Analysis

**Figure 4:**
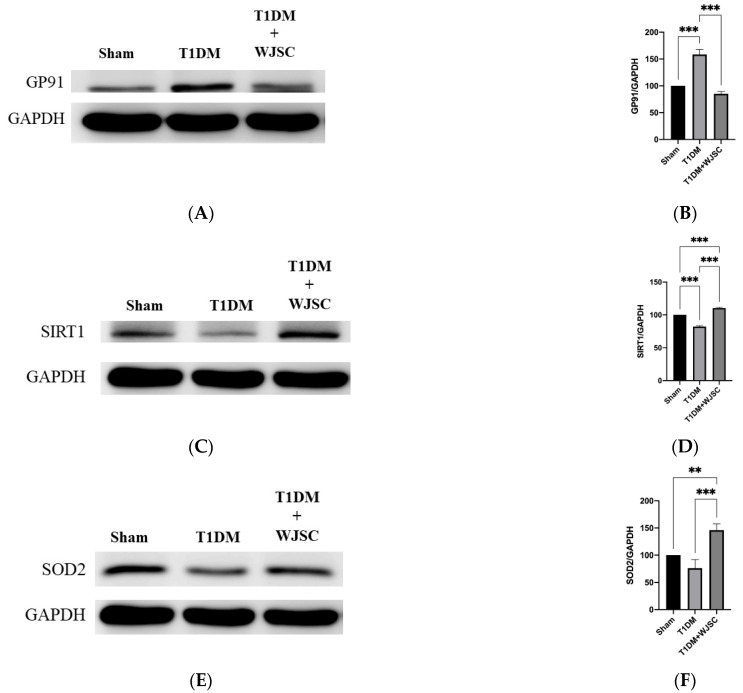
Exploring oxidative stress related markers for heart tissues using western blotting analysis. (**A**) GP91 protein expression; (**B**) quantification of GP91 expression; (**C**) Sirt-1 protein expression; (**D**) quantification of Sirt-1 expression; (**E**) SOD2 protein expression; (**F**) quantification of SOD2 expression. ** *p* < 0.01; *** *p* < 0.001.

Protein expression was analyzed using western blot. The blotting band intensity is positively associated with the protein expression level. Figure 4A illustrates GP91 protein expression for experimental heart tissues. GP91 expression quantification for the Sham, T1DM and T1DM + WJSC follows the order 100 ± 0, 159 ± 9 and 85 ± 4, respectively (Figure 4B). Significance can be found between the Sham vs. T1DM (Sham < T1DM, *p* < 0.001) and T1DM vs. T1DM + WJSC (T1DM > T1DM + WJSC, *p* < 0.001). Figure 4C states antioxidant marker Sirt-1 expression for experimental heart tissues. The Sirt-1 expression quantification for the Sham, T1DM and T1DM + WJSC follows the order 100 ± 0, 82 ± 1.4 and 111 ± 1.0, respectively (Figure 4D). Statistical significance can be observed between the Sham vs. T1DM (Sham > T1DM, *p* < 0.001), T1DM vs. T1DM + WJSC (T1DM < T1DM + WJSC, *p* < 0.001) and Sham vs. T1DM + WJSC (Sham < T1DM + WJSC, *p* < 0.001). Figure 4E points out antioxidant protein marker SOD2 expression for experimental heart tissues. SOD2 expression quantification for the Sham, T1DM and T1DM + WJSC follows the order 100 ± 0, 76 ± 16 and 146 ± 12, respectively (Figure 4F). Significance can be found between the Sham vs. T1DM + WJSC (Sham < T1DM + WJSC, *p* < 0.01) and T1DM vs. T1DM + WJSC (T1DM < T1DM + WJSC, *p* < 0.001).

### 3.4. Investigating Inflammasome Related Protein Expression Using Western Blotting Analysis

**Figure 5:**
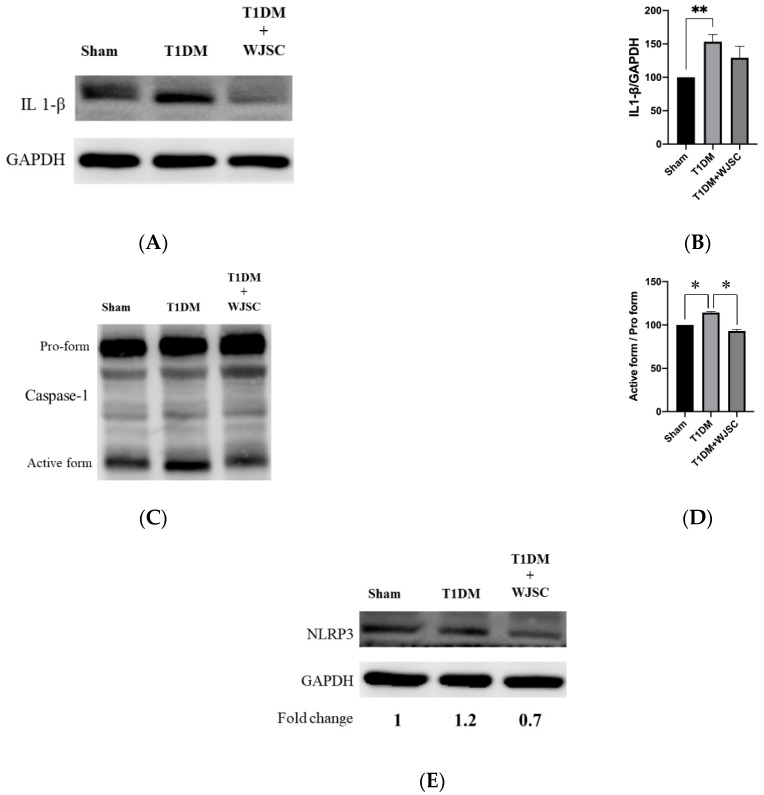
Exploring inflammasome related markers for heart tissues using western blotting analysis. (**A**) IL-1β protein expression; (**B**) quantification of IL-1β expression; (**C**) Caspase-1 protein expression; (**D**) quantification of caspase-1 expression; (**E**) NLRP3 protein expression. * *p* < 0.05; ** *p* < 0.01.

Several main proteins are involved in inflammasome signaling activation, including IL-1β, caspase-1 and NLRP3. Figure 5A illustrates IL-1β expression for experimental heart tissues. The IL-1β expression quantification for the Sham, T1DM and T1DM + WJSC follows the order 100 ± 0, 153 ± 11 and 129 ± 17, respectively (Figure 5B). Statistical significance is shown between the Sham vs. T1DM (Sham < T1DM, *p* < 0.01). Figure 5C shows caspase-1 expression for experimental heart tissues. The pro-caspase-1 vs. active-caspase-1 ratio for the Sham, T1DM and T1DM + WJSC follows the order 100 ± 0, 118 ± 3 and 94 ± 5, respectively (Figure 5D). Significance can be observed between the Sham vs. T1DM (Sham < T1DM, *p* < 0.05) and T1DM vs. T1DM + WJSC (T1DM < T1DM + WJSC, *p* < 0.05). Figure 5E indicates the expression of another inflammasome related protein marker, NLRP3. The fold change for the Sham, T1DM and T1DM+WJSC follows the order 1, 1.2 and 0.7, respectively.

## 4. Discussion

A diabetic (or high glucose) environment is capable of triggering various pathological signals. Pathological signal activation is greatly associated with cardiomyocyte damage, leading to diabetic cardiomyopathy progression. From the experimental findings, we find that diabetes induces structural changes (Figure 1), fibrotic collagen deposition (Figure 2) and ROS production (Figure 3) in heart tissue. Meanwhile, molecular changes indicate that inflammasome related protein expression including IL-1 beta (Figure 5A), caspase-1 (Figure 5C) and NLRP3 (Figure 5E) can be observed in diabetic heart tissues. These findings reveal that diabetic cardiomyopathy progression involves structural changes, fibrosis, ROS production and inflammasome related protein expression in heart tissue. These results are consistent with a previous study [17,18]. This study performed stem cell-based therapy as one of the therapeutic strategies in the treatment of diabetic cardiomyopathy. After mesenchymal stem cell transplantation, all pathological conditions described above were significantly improved. These results show that mesenchymal stem cell transplantation shows potential in the treatment of diabetic cardiomyopathy through oxidative stress regulation and the NLRP3 inflammasome pathway.

As previously mentioned, although stem cells show a therapeutic effect on diabetic cardiomyopathy, the mediators between stem cells, inflammasome and diabetic cardiomyopathy remain unclear. Zhang et al. [19] illustrated that ROS production and inflammasome related proteins (NLRP3, IL-1 beta and caspase-1) can be observed in H9c2 cardio myoblast damage induced by a high glucose environment. ROS neutralization subsequently reduced inflammasome related protein expression. Qu et al. [20] provided similar evidence to confirm that ROS reduction by antioxidant oral administration is capable of suppressing inflammasome related protein expression, leading to improved diabetic cardiomyopathy. The aforementioned indicates that a diabetic environment first induces ROS production, and second inflammasome pathway activation. Therefore, ROS production plays the upstream role and inflammasome pathway activation acts as the downstream role in diabetic cardiomyopathy progression. From our data, we see that GP91 protein expression was found in the diabetic group (T1DM in Figure 4A). GP91 protein suppression can be observed in the treatment group (T1DM + WJSC in Figure 4A). These findings suggest that diabetic environment can increase GP91 protein expression in heart tissues. Stem cell transplantation can suppress GP91 protein expression. We know that GP91 is one of the proteins located in the cell membrane. GP91 expression induces superoxide production, leading to increased cellular ROS. Thus, our data implies that a diabetic environment increases ROS production through GP91 protein expression. ROS production then activates the inflammasome pathway, resulting in diabetic cardiomyopathy progression. Stem cells transplantation can suppress the pathological signaling induced by GP91/ROS/inflammasome axis, contributing to improved diabetic cardiomyopathy. Zhang et al. [21] state that upregulation of NOX2/gp91 can be observed in rats with renal ischemia-reperfusion injury. Injection of microvesicles (MVs) derived from human Wharton’s Jelly mesenchymal stromal cells can alleviate renal ischemia-reperfusion injury in rats because MVs contain lots of biomolecules, including miRNAs which can block mRNA of NOX2/gp91, leading to downregulation of NOX2/gp91.

In addition to blocking GP91, we find that antioxidant proteins, as well as Sirt-1 and SOD2 expression, also play an important role in ameliorating diabetic cardiomyopathy progression through scavenging ROS production. Chen et al. [22] pointed out that adipose-derived stem cells transplantation can increase Sirt-1 expression in heart tissues under diabetic environment, and Sirt-1 expression then scavenges ROS production, leading to improved diabetic cardiomyopathy. In this study, a diabetic environment reduces expression of antioxidant proteins, as well as Sirt-1 and SOD2 expression (Figure 4C,E). Elevated oxidative stress then activates the inflammasome pathway, leading to diabetic cardiomyopathy progression. Stem cell transplantation restores Sirt-1 and SOD2 expression, resulting in ROS/inflammasome signaling blocking and diabetic cardiomyopathy improvement. Furthermore, Hu et al. [23] reveal that paracrine secretion of IGF-1 from human Wharton’s Jelly mesenchymal stromal cells can activate expression of SOD2 in damaged H9c2 cardiomyoblasts, resulting in recovery of H9c2 cells. These studies imply that secretion of MVs or IGF1 protein from mesenchymal stem cells play important roles in the treatment of damaged cells.

Although the ROS/inflammasome axis is suggested to be involved in the progress of diabetic cardiomyopathy, there are some limitations that should be considered in this study. Firstly, the regulatory roles of inflammasome signal should be further investigated. For instance, the use of siRNAs (such as siNLRP3) to silence inflammasome markers in cell models or the use of transgenic mice to confirm the above observations. Secondly, how WJSCs regulate GP91/inflammasome axis should be elucidated. For instance, microvesicles or exosomes derived from WJSC may be potential candidates to involve in the treatment of diabetic cardiomyopathy.

## 5. Conclusions

This paper provides novel evidence to confirm that mesenchymal stem cells transplantation exerts a therapeutic effect on diabetic cardiomyopathy through GP91/ROS/inflammasome axis regulation (graphic summary). These findings also provide an alternative treatment for patients with diabetic cardiomyopathy.

## Data Availability

The data that support the findings of this study are available from the corresponding author upon reasonable request.
